# Patient’s expectations and perceptions on quality of care; An evaluation using SERVQUAL gap in Kenya

**DOI:** 10.1371/journal.pone.0315910

**Published:** 2025-03-04

**Authors:** Agnes Karingo Karume, Kelvin Nyongesa, Lydia Okutoyi, John Kinuthia

**Affiliations:** 1 Research & Programs Department, Kenyatta National Hospital, Nairobi, Kenya; 2 Division of Healthcare Quality, Kenyatta National Hospital, Nairobi, Kenya; University of Sharjah College of Health Sciences, UNITED ARAB EMIRATES

## Abstract

**Background:**

Assessing healthcare quality is pivotal for patient-centric enhancements. Employing the Gaps Model of Service Quality and SERVQUAL, this study determined patient expectations and perceptions of care quality at a national referral hospital.

**Methods:**

In this cross-sectional study, data were gathered pre and post care using a semi-structured tool with 380 participants. Statistical package for social sciences(Version 25) facilitated analysis. Quality indices, expressed as percentages of weighted averages, compared expected and perceived care. Service quality gaps, which represented the difference between perception and expectation (P – E), were assessed via paired-sample t-tests. Logistic regression were used to examine factors linked to positive service quality gaps.

**Results:**

Median age was 34 years, 74% were female, 57% had non-formal employment, and 49% had attained secondary education. The gap index across all components was -10%. The largest gaps existed in SERVQUAL’s reliability and empathy domains. Secondary education (OR = 2.13, 95% CI: 1.01–5.21,p = 0.003; aOR =  2.0, 95%CI: 1.57–7.11) and non-formal employment (OR = 1.54, 95% CI: 1.01–4.11, p = 0.01; aOR =  1.40, 95%CI: 1.08–5.12) correlated with positive health service quality gaps.

**Conclusions:**

Patient expectations exceeded perceptions of care quality, emphasizing the need to align service delivery and actively manage patient expectations, particularly in areas of reliability and empathy, such as timeliness and compassion.

## Introduction

The Health Systems Framework established by the World Health Organization (WHO) recognizes the pivotal role of Quality and Safety as intermediaries, essential for achieving desirable health outcomes such as enhanced health and responsiveness [1]. In recent times, there has been a growing acknowledgment and utilization of understanding and measuring patient expectations and perceptions. This understanding is central to the evaluation of patient satisfaction and the delivery of patient-centered care [2].

The perception of quality care pertains to an individual’s personal viewpoint on the services they receive, while expectations encompass the elements one anticipates being associated with the service. Oliver’s Expectation Confirmation Model of 1980 [3], posits that when a service surpasses expectations (positive disconfirmation), it leads to satisfaction. Conversely, if it falls short of expectations (negative disconfirmation), it is likely to result in dissatisfaction [4]. Notably, patients’ expectations and perceptions regarding the quality of care significantly influence their level of satisfaction. This, in turn, has far-reaching consequences, including their choice of healthcare providers, the retention of customers or patient loyalty, the dissemination of positive or negative feedback, employee satisfaction, productivity, and the overall profitability of healthcare institutions [5–7].

Patients’ expectations and perceptions of the quality of care paint a dynamic picture of contemporary healthcare practice and provision. Striking a balance between the two is imperative to remain competitive. Despite the commonly held belief that public healthcare lags behind private healthcare in terms of quality, there is a dearth of studies in this particular field [8]. Given the heightened focus on quality within the public sector, it is crucial to evaluate whether these efforts align with customer expectations and perceptions of care [1].

Patient well-being in a healthcare context can be defined by five key components that transcend various healthcare departments: the reliability of care delivered, assurances provided, tangibles available, responsiveness to care needs, and empathy towards patient needs. These components were originally identified in the SERVQUAL model by Parasuraman, Valarie A. Zeithaml, and Leonard L. Berry [9]. However, they have undergone adaptations and applications in diverse global settings to investigate the quality of care [10].Notably, differences between expected and perceived service quality often arise depending on the context [11]. While several studies have delved into expectations and perceptions in specific disease contexts, there is a growing movement towards encompassing various clinical and non-clinical contexts for a comprehensive understanding of the entirety of the patient experience. This underscores the need for standardized assessments of patient expectations [6,11].

Numerous initiatives have been undertaken to enhance the quality of care, including efforts to streamline referrals, the establishment of healthcare quality divisions, and the adoption of the Kenya Quality Model for Health at the national level—a conceptual framework for integrated coordination. However, there remains a scarcity of data to ascertain whether these concerted efforts align with patient expectations and pinpoint specific areas for improvement. Existing studies have predominantly examined the quality of care within the scope of specific disease processes, leaving a substantial gap in the assessment of a critical aspect of care: the overall care experience beyond specific clinical treatments [12,13].

Furthermore, mounting evidence suggests that clients may assign varying degrees of importance to different facets of quality provision and that their perception of quality ultimately hinges on their expectations [9,14]. This aspect has yet to be clearly defined within the healthcare context in Kenya and, more specifically, within the national referral hospital, which caters to patients from diverse regions of the country.

Anecdotal evidence from hospital-based quality assessments and patient satisfaction surveys has indicated disparities in the quality of care experienced by patients seeking treatment at the facility. This compellingly underscores the necessity to investigate both the expectations and perceptions concerning the quality of care rendered. The findings from this study will play a pivotal role in enabling leadership and management to enact policies aimed at addressing underlying gaps in care. Moreover, a standardized approach, akin to that employed in this study, could be adopted to assess healthcare service quality across the region.

## Methodology

### Study design

This study adopted a cross-sectional design, involving data collection at two distinct time points: pre-care and post-care. Patients seeking healthcare services at the hospital outpatient department were approached for participation. The study recruited patients both from the outpatient setting and those subsequently admitted for inpatient care following an initial outpatient review. Data was collected at two junctures: prior to the commencement of care and after the completion of care. For those exclusively receiving outpatient services, data was collected before and after outpatient care, whereas for patients transitioning to inpatient care, data was collected before admission and following discharge from the inpatient ward. The investigation utilized a semi-structured questionnaire to assess the quality of care, drawing upon the SERVQUAL tool, and to evaluate the disparities between perceived and expected care.

### Study setting

The study took place within the outpatient clinics and inpatient wards encompassing medical, surgical, obstetrics departments,between October 2020 to December 2020. Notably, this hospital holds the prestigious status of being the foremost referral hospital in Kenya, receiving patients referred from various lower-tier healthcare facilities across the country. The majority of patients referred to this institution seek specialized care.

### Study subjects and sampling

A total of three hundred and eighty patients meeting the defined inclusion criteria were enrolled consecutively in the study over a duration of two and a half months, spanning from October to December 2020, until the predetermined sample size was achieved. This encompassed clinically stable patients of sound mental competence who were above the age of 18 years. Additionally, primary caretakers of patients below the age of 18 years were included in the study. Inpatients integrated into the study had spent a minimum of two nights in the hospital wards to facilitate a comprehensive assessment of the services rendered. The determination of the sample size was based on the monthly attendance figures, segmented across four clinical areas: obstetrics and gynecology, pediatrics, medicine, and surgery, for both outpatient and inpatient settings.

The study targeted the population of interest, which comprised patients seeking care within the outpatient and inpatient settings of the specified medical departments. On average, each department admitted 20 patients daily, translating to a monthly total of 2,240 admissions across all departments. In parallel, each outpatient clinic received an average daily attendance of 50 patients. Table 1 provides an overview of the target population and the aggregated sample size.

**Table 1:** Target population.

Site	Target Population	Aggregate Sample
Medical Outpatient	1400	1400/7840 * 380 = 68
Medical InpatientInpatient	560	27
Surgical Outpatient	1400	68
Surgical InpatientInpatient	560	27
Obstetric Outpatient	1400	68
Obstetric InpatientInpatient	560	27
Pediatric Outpatient	1400	68
Paediatric InpatientInpatient	560	27
Total	7840	380

### Sample size determination

Using Yamane Taro (1967) formula [15]:


$$\text{n=N/}\left(\text{1+N}{\left(\text{e}\right)}^{\text{2}}\right)$$

Where: n = sample size

N = target population

e= margin of error (95% confidence interval)

The sample size was thus:


$$\text{n=7840/(1+7840(0.0}{\text{5}}^{\text{2}}\text{)}$$

=380 patients

### Sampling technique

A simple random sampling technique was employed in the selection of participants for this study. Each patient within the study units was assigned a unique identifier during the data collection process. Subsequently, all patients who selected odd-numbered identifiers were included as participants in the study.

### SERVQUAL instrument

To assess the quality of health services, the study utilized the Gaps Model of Service Quality in conjunction with the SERVQUAL instrument. This instrument facilitated the measurement of the gap between patient perception and expectation across five crucial components: reliability, tangibles, assurance, responsiveness, and empathy. These components were originally delineated by Parasuraman, Valarie A. Zeithaml, and Leonard L. Berry [9]. In the evaluation of care quality, international SERVQUAL standards were referenced, and the specific questions were tailored to the hospital’s context while retaining the individual SERVQUAL component questions. To gauge internal consistency, Cronbach’s alpha was computed, with all components exhibiting values exceeding the recommended threshold of 0.7 [16].

Consequently, the questionnaire encompassed demographic information, including age, gender, level of education, employment status, and the five dimensions of care quality. Participants were asked to rate these dimensions on a 5-point Likert scale, ranging from “strongly disagree” to “strongly agree.” Higher scores on each item indicated that patients held more favorable expectations and perceptions regarding the quality of services. In each section, an additional open-ended question was included to provide insights into the responses given. The same set of questions was posed both before and after the receipt of care within the hospital.

### Study procedures

Data collection was conducted over a period of three months, spanning from October to December 2020. By this time, most COVID-19-related movement restrictions in Kenya had been lifted, allowing for more flexible operations in hospitals and clinics. Despite the easing of restrictions, we adhered to all necessary COVID-19 safety protocols as mandated by the Ministry of Health, including the use of personal protective equipment (PPE), social distancing, hand hygiene measures, and limiting in-person interactions where possible. These measures ensured that data collection was conducted safely while maintaining the integrity of the study. The data collection tool was administered through interviews conducted in the English language. To assist in the administration of the study tool, the researcher trained and enlisted the support of two qualified medical students as research assistants.

Prior to participation, eligible individuals were provided with a comprehensive explanation of the consent form, ensuring that they fully comprehended its contents. Only those who voluntarily consented were enrolled in the study. Data collection commenced with the acquisition of demographic information, followed by the assessment of participants’ expectations before the administration of healthcare services. The section related to perceptions was completed either in the clinic following service receipt or after discharge from the inpatient wards, as applicable to the specific respondents’ circumstances.

### Reliability

Reliability analysis was conducted to assess the internal consistency of the five domains under examination. The results indicated that all five domains demonstrated a high level of reliability, with Cronbach’s alpha (α) values exceeding the recommended threshold of 0.7 [17]. These findings are summarized in Table 2 [16]. as shown in Table 2.

**Table 2:** Reliability of the scores.

	Cronbach alpha value
Reliability	0.79
Assurance	0.85
Responsiveness	0.87
Tangibles	0.91
Empathy	0.88

### Outcome variable

Service quality was assessed based on the questions within the SERVQUAL instrument, and the quality of care gap was calculated as follows: Quality of Care Gap =  Perception Score - Expectation Score. To derive the overall SERVQUAL score, the average of the gap scores across all five dimensions was computed using the formula: Overall SERVQUAL Score =  (Gap Tangibles +  Gap Reliability +  Gap Responsiveness +  Gap Assurance +  Gap Empathy)/ 5. A negative difference was indicative of a decrease in quality, while a positive difference score signified a positive perception of service quality. The dependent (outcome) variable is the overall measure of service quality, derived from the composite SERVQUAL score across all domains for each respondent. This composite score is created by summing the individual scores within each domain, capturing a holistic view of service quality for each participant.

For analysis, we categorize service quality as follows:

- Positive Service Quality (1): An overall positive SERVQUAL score, indicating that the participant’s perceptions of service exceeded their expectations, thus indicating satisfactory or high-quality service.
- Negative Service Quality (0): An overall negative SERVQUAL score, where expectations were not met.

This categorization allows us to determine whether participants perceive the service quality positively or negatively, based on a combined assessment across all SERVQUAL domains.

### Data analysis

The data analysis was conducted using the Statistical Package for the Social Sciences (SPSS). A combination of descriptive and inferential statistics was employed, with a particular focus on comparisons across the five SERVQUAL domains to ascertain the expectation-perception gap. Demographic characteristics were analyzed descriptively, employing frequencies and percentages.

Service quality, the outcome variable, was categorized as positive or negative based on the difference between expectations and perceptions. Paired-sample t-tests were utilized to compare the expectations and perceptions of service quality, enabling the identification of services characterized by the most significant gaps in quality.

In the analysis, the independent variables encompassed components of the SERVQUAL tool, while the dependent variable was the gap in health service quality. Binary logistic regression analysis was employed to explore the factors associated with positive gaps in health service quality. Odds ratios (OR) with corresponding 95% confidence intervals (CI) were computed to assess the strength of associations. The independent variables in this analysis included demographic characteristics, while the dependent/outcome variable was the service quality..

A significance level of 0.05 was adopted, whereby variables with p-values less than 0.05 were deemed statistically significant. Variables with p-values less than 0.05 were included in the multivariable analysis model to explore potential predictors of positive health service quality gaps.

### Ethical consideration

The study adhered to ethical standards and obtained approval from the hospital’s Research and Ethics Committee, as evidenced by the ERC Number: P76/02/2020. Participants were fully informed about their rights and autonomy in deciding whether to participate in the study, emphasizing their absolute freedom to withdraw from the study at any point without incurring any adverse consequences.

Patients who met the predefined inclusion criteria were provided with a comprehensive explanation of the study’s objectives and procedures. They were guided through the informed consent process, and only those who voluntarily provided their informed consent were enrolled as participants in the study.

To ensure the privacy and confidentiality of participants, their identities and personal information were kept strictly anonymous, with no recording of their names or identifying details on the questionnaires. Participants continued to receive their healthcare and follow-up in the standard manner, and their participation in the study did not result in any undue disadvantages or alterations to their care.

## Results

### Patient characteristics

Among the 380 participants, 74% (n =  282) were female, 26% (n =  98) were male, and the median age was 34 (Table 3). Approximately 49% (n =  186) had completed secondary education, while 31% (n =  118) had primary education. In terms of employment, 57% (n =  215) engaged in non-formal work, 28% (n =  105) had formal employment, and 16% were unemployed (n =  60).

**Table 3:** Demographic characteristics of the study participants.

Demographic factors	Frequency	Percent(%)
**Gender**		
Male	98	26
Female	282	74
**Age, Median (IQR)**	34(28 - 43)	
**Patient type**		
Outpatient	228	60
InpatientInpatient	152	40
**Highest level of education**		
Not studied	10	3
Primary	118	31
Secondary	186	49
Certificate	29	8
Diploma	23	6
Undergraduate	13	3
Masters degree	1	0
**Source of income**		
Formal employment	105	28
Non-formal employment	216	57
Unemployment	59	16
**First time receiving care at the facility**		
Yes	304	80
No	76	20
**Finding a way to the hospital**		
I was referred by another facility	281	74
I was referred by a friend or colleague	4	1
I came on my own volition	85	22
Missing	10	3

The majority sought outpatient care (60%, n =  228), with 40% being inpatients (n =  152). First-time clients constituted 80% (n =  304). Referral status revealed 74% (n =  281) referred from other facilities, 22% (n =  84) came voluntarily, 1% (n =  4) were referred by acquaintances, and 3% (n =  11) arrived due to accidents or trauma.

Overall, 17% (66/380) reported positive quality of care gaps.

### Patient’s expectations and perceptions of the quality of provided services

The comprehensive expectation index scored at 93.00%, contrasted with the perception index, which achieved 83.30%. Consequently, the overall gap between perception and expectation amounted to -9.70%. Notably, the most pronounced gaps between perception and expectation were observed in the domains of reliability (15.58) and empathy (11.23). (Refer to Table 4 for Expectation-Perception Indexes.)

**Table 4:** Expectation-perception gap indexes.

Components	Expected index (E) (%)	Perceived Index (P) (%)	Gap (P – E)
Reliability	90.23	74.65	-15.58
Assurance	94.53	88.38	-6.15
Tangibles	93.33	83.95	-9.38
Responsiveness	95.18	89.23	-5.95
Empathy	91.56	80.33	-11.23
Overall	93.00	83.30	-9.70

### Comparison of expectation-perception gap based on the SERVQUAL components

In this analysis, the independent variables comprised various components of the SERVQUAL tool, while the dependent variable was the gap in health quality. Notably, there was a statistically significant difference observed in the quality of care gap among the SERVQUAL components (p <  0.05). Specifically, positive gap differences were identified in the domains of environmental cleanliness (+0.21), environmental visual appeal (+0.01), staff neatness (+0.18), and the practice of addressing patients by their names (+0.35)(Table 5)

**Table 5:** Comparison of expectation-perception gap based on the SERVQUAL components.

Components	Gap(Perceived - Expected)	t-statistic	p-value
**Reliability**			
Speed of services	-1.33	-3.78	<0.001
Length of waiting times	-1.72	-4.0	<0.001
Explanation of care plans	-0.16	-3.73	<0.001
Questions addressed	-0.20	-5.32	<0.001
**Assurance**			
Expert help and definitive treatment	-0.08	-2.94	0.003
Privacy during care	-0.85	-5.74	<0.001
Information kept confidential	-0.07	-7.39	<0.001
Receive all the treatment needed	-0.26	-4.02	<0.001
**Tangibles**			
The environmental cleanliness	^+^0.21	-4.56	<0.001
The food cooked	-1.47	-4.91	<0.001
The environment’s visual appeal	^+^0.01	-4.75	<0.001
The staff neatness	^+^0.18	-5.60	<0.001
**Responsiveness**			
Assistance from staff when needed	-0.35	-2.42	0.02
Staff readiness and willingness to explain concerns	-0.30	-3.25	0.001
Communication on medical condition and care	-0.16	-4.63	<0.001
Information on the timing of services	-0.08	-3.96	<0.001
**Empathy**			
Address using name	^+^0.35	-5.89	<0.001
Introduction of person giving care	-2.12	-6.63	<0.001
Humane treatment	-0.16	-3.11	0.002
Staff politeness and courteousness	-0.34	-3.11	0.01

### Factors associated with the negative gap between expectation and perception

In our analysis, demographic characteristics served as independent variables, while the dependent variable was categorized as either a positive or negative health service quality gap.

The results from binary logistic regression bivariable analysis revealed significant associations. Participants with secondary education were 2.1 times more likely to experience a positive health service quality gap than those with primary education (OR =  2.13, 95% CI: 1.01 - 5.21, p =  0.003). Similarly, individuals with tertiary education were 3.4 times more likely to report a positive health service quality gap compared to those with primary education (OR =  3.41, 95% CI: 1.67 - 6.78, p <  0.001). Non-formal employment was associated with a 1.5-fold higher likelihood of a positive health service quality gap compared to unemployment (OR =  1.54, 95% CI: 1.01 - 4.11, p =  0.009).

These significant findings were reaffirmed in the multivariable analysis, adjusting for all relevant factors. Secondary education was linked to twice the likelihood of experiencing a positive health service quality gap compared to primary education (aOR =  2.0, 95% CI: 1.57 - 7.11, p =  0.013). Furthermore, individuals engaged in non-formal employment remained 1.4 times more likely to encounter a positive health service quality gap than the unemployed (aOR =  1.40, 95% CI: 1.08 - 5.12, p =  0.016). (Table 6)

**Table 6:** Demographic factors associated with negative gap between expectation and perception.

Demographic characteristics	Service quality					
	Negative gap n (%)	Positive gap	OR(95%CI)	p-value	aOR(95%CI)	P-value
		n (%)				
**Referral**						
I was referred from another facility	45(68)	236(78)	2.11(0.56 - 5.11)	0.01		
I was referred by a friend or colleague	2(3)	2(1)	0.87(0.33 - 2.61)	0.06		
I came on my own volition	19(29)	66(22)	1			
**Gender**						
Male	16(24)	82(26)	0.45(0.21 -2.11)	0.44		
Female	50(76)	232(74)	1			
**Age of patient**						
Less than 30 years	24(36)	95(31)	1			
30 - 49 years	29(44)	168(54)	0.56(0.23 - 1.21)	0.32		
50 years and above	13(20)	48(15)	1.31(0.33 - 2.12)			
**Highest level of education**						
Not studied	3(5)	7(2)	1			
Primary	11(17)	107(34)	1.13(0.31 – 3.11)	0.23		
Secondary	34(52)	152(48)	2.13(1.01 - 5.21)	0.003	2.0(1.57 - 7.11)	0.01
Tertiary	18(27)	48(15)	3.41(1.67 - 6.78)	<0.001		
**Source of income**						
Formal employment	28(42)	77(25)	0.51(0.22 - 2.12)	0.51		
Non-formal employment	32(49)	184(59)	1.54(1.01 - 4.11)	0.01	1.40(1.08 - 5.12)	0.02
Unemployment	6(9)	53(17)	1			
**First time at the facility**						
Yes	54(82)	250(80)	0.16(0.11 - 2.31)	0.42		
No	12(18)	64(21)	1			

## Discussion

### Overall service quality gap

Our analysis reveals a notable dissonance between patients’ expectations for healthcare service quality at the hospital and their actual perceptions. Patients arrived with high expectations, which often diverged from the reality of the care they received. Across all dimensions of service quality assessed by the SERVQUAL tool, with the exception of a few specific components within the tangibles and empathy categories, patients’ perceptions consistently signaled dissatisfaction in relation to their initial expectations. The presence of a negative service quality gap implies that customer expectations for quality have not been met. These findings align with those of several analogous studies conducted in similar contexts, where patients exhibited a diminished perception of care quality when comparing their expectations to their actual experiences, resulting in a negative quality of care gap [5–7,18]. For example, research in Damascus hospitals revealed negative gaps across SERVQUAL dimensions, except tangibles, similar to our findings. Patients expressed the highest expectations for empathy and responsiveness, yet the largest dissatisfaction stemmed from interactions and wait times, particularly the perceived lack of attentiveness and time spent with patients by staff [18,19].These findings support the general trend of high patient expectations for empathy in healthcare, a dimension often under-met due to structural constraints in many public hospitals in low-resource settings. A negative service quality gap highlights the critical need for healthcare managers and policymakers to address patient-identified areas of dissatisfaction, aligning service improvements with patient priorities and systematically gathering feedback to optimize resource allocation for the greatest impact.

### Tangibles and service quality

In the realm of tangible components, patients consistently exhibited higher perceptions compared to their initial expectations. Patients’ expectations pertaining to overall environmental cleanliness, visual appeal of the surroundings, and the neatness of staff attire were not only met but exceeded, as evidenced by higher perception scores. This finding aligns with studies in the UAE and Jordan, where patients similarly reported high satisfaction in tangibles [18,19]. One pivotal reason for the significance of the physical environment is its profound influence on patients’ initial impressions and expectations. The physical surroundings play a crucial role in shaping patients’ preliminary judgments about service quality, which subsequently informs their overall perceptions of the service [18]. The physical environment can significantly impact customers’ comfort, convenience, and functionality during their engagement with the service. Previous research has demonstrated that attributes such as cleanliness, comfort, and aesthetic appeal contribute significantly to the overall perceived quality of a service [9]. These findings underscore the pivotal role of the tangible dimension in mitigating deficiencies observed in other facets of service quality.

### Empathy and service quality

A notable finding is that patients consistently appreciated being addressed by name, which strengthens perceived empathy and aligns with culturally significant expectations for respectful interaction. Addressing patients using their names serves as a simple yet highly effective means of establishing a personalized connection and augmenting the patient-provider relationship [20]. Previous research has substantiated the positive impact of patients being addressed by name, as it correlates with higher satisfaction levels, increased perceptions of provider empathy, and improved perceived understanding of patients’ needs [20,21]. It is imperative however, to underscore the necessity of accompanying patient names with unique identifiers to avert potential medical errors resulting from similarities in names. In contrast, patients’ expectations in other dimensions of empathy were not consistently fulfilled, notably with the infrequent introduction of healthcare providers. Similar findings are seen in an Iranian hospital whereby not all clinicians introduced themselves [22]. The act of introducing oneself holds substantial significance, as it instills confidence in the provider and positively influences the perception of the quality of care being delivered. A negative expectation perception gap was additionally seen in other aspects of empathy such as humane treatment and staff politeness and courteousness. In the study by Nadi et al. [23], empathy emerged as the most critical aspect within SERVQUAL, highlighting its significance in patient expectations. High expectations regarding empathy have similarly been observed in other studies [24,25], where empathy is associated with providing individualized attention to patients’ concerns.

### Reliability and service quality

Our study showed a pronounced gap in reliability, specifically regarding waiting times and service efficiency. Patients expressed dissatisfaction with the waiting times and perceived the speed of services as slower than their initial expectations. This perception of extended waiting and slower service may convey a sense of inadequate attention to their needs, potentially undermining trust and contributing to an unfavorable overall perception of care quality. Effectively managing patient expectations regarding the speed and timeliness of services is a challenging task, as evidenced by similar studies that have revealed substantial gaps between patient perceptions and expectations, particularly in the dimension of reliability [18,26]. In Kenya, referral hospitals often face high patient volumes, further straining resources and impacting service speed. Enhanced appointment systems and better communication on wait times may help manage patient expectations and improve satisfaction in the reliability domain.

### Assurance and service quality

Patients felt assured of the expertise available at this national referral hospital. Most patients were assured they would have expert help and definitive treatment in the facility. This aligns with findings from Tanzanian referral hospitals, where assurance was rated highly due to patients’ confidence in receiving specialized care [27]. Customers’ knowledge and confidence in the services they are about to receive or have received helps build trust and creates a positive perception of the service provider [18]. However, the teaching environment in this facility sometimes disrupts privacy, an aspect negatively perceived by patients in a similar studies in referral hospitals [28–30]. Balancing educational needs with patient privacy considerations is essential to maintain high perceived assurance. Other studies have identified assurance as the highest-scoring domain compared to others [31,32] whereby assurance is associated with the availability of competent, trained, and qualified staff.

### Responsiveness and service quality

The perception of service quality by patients is intricately linked to the willingness and timeliness with which healthcare providers accommodate their needs [33]. In this study, it is noteworthy that patients’ expectations regarding the empathetic aspects of care surpassed their actual experiences, thus indicating a level of dissatisfaction. Responsiveness had the greatest impact on customer satisfaction in a study conducted across four hospitals in Jordan [34]. However, in a similar study in the UAE, responsiveness received the lowest scores among SERVQUAL dimensions, with public facilities scoring lower than private ones [14]. It is important to acknowledge that external factors, such as provider workload and resource constraints, which are often encountered in publicly funded healthcare facilities, could potentially hinder the capacity of providers to offer personalized assistance and effectively respond to patient concerns [35–37]. While our research did not delve into these external factors, it underscores the imperative for future investigations to explore strategies that enable healthcare providers to better align with and respond to the diverse needs of their patients.

### Individual factors that may affect quality of care expectations and perceptions

Education level, income, and the frequency of visits have previously been seen as important sociodemographic factors which may determine the patient’s expectations and perception of the quality of services provided [18]. Patients with a higher level of education exhibited a more favorable perception of the quality of care than expected. It is possible that patients with higher levels of education are aware of the challenges faced by major referral hospitals in developing countries, particularly due to the high patient volume, which limits the capacity to deliver high-quality care. This awareness may lower their initial expectations. These findings align with previous studies that have shown a link between a patient’s level of education and their perception of care quality [8,38].

Although prior hospital experiences were thought to influence the service quality gap, our study did not find a statistically significant difference in this regard. This contrasts with studies that found a positive association between prior knowledge about care delivery in a healthcare facility and the perceived service quality gap [17,18]. Further research is needed to thoroughly assess the impact of socio-demographic factors on service expectations and perceptions.

### Limitations

This study specifically focuses on the expectations and perceptions of quality of care in a public national referral hospital. It is worth noting that these findings may not be directly applicable to private healthcare facilities, which are often recognized for their heightened emphasis on tangible elements such as visual appeal. Consequently, the generalizability of our results beyond the public healthcare sector may be limited.

Furthermore, it is important to acknowledge that the cross-sectional nature of our analysis restricts our ability to establish causal relationships between differences in perceptions and expectations regarding the quality of care.

## Conclusion

In all five dimensions of SERVQUAL, our analysis revealed negative gaps between the expectation and perception indexes, indicating that patients did not attain full satisfaction with the quality of healthcare services. This study serves as a valuable platform for discerning both the strengths and weaknesses in healthcare quality. Efforts must be directed toward optimizing service delivery and proactively managing patients’ expectations, with a particular emphasis on enhancing reliability factors such as service timeliness and empathy. By addressing these areas, healthcare providers can work towards elevating the overall quality of care and better meeting the needs of their patients.

## Recommendation and implication for future studies

The results derived from this study underscore the imperative of instituting a systematic framework for the ongoing monitoring and assessment of healthcare service quality. This framework should encompass the continuous collection and utilization of feedback from both patients and staff, in conjunction with the utilization of quality indicators. These measures will facilitate the identification of areas requiring enhancement and the subsequent implementation of quality improvement initiatives. Moreover, tracking and documenting progress over time will be pivotal in ensuring the sustained advancement of healthcare service quality. Subsequent research endeavors should delve deeper into the development and refinement of such monitoring systems to further enhance the quality of healthcare services.

## Supporting information

S1 File Questionnaire-Expectations and perception. (PDF)

S1 Dataset Expectation and perception. (XLSX)
