# Supplementary material for: Patient’s expectations and perceptions on quality of care; An evaluation using SERVQUAL gap in Kenya
Source: PLoS One. 2025 Mar 4;20(3):e0315910. doi: 10.1371/journal.pone.0315910 (PMC11878910; doi:10.1371/journal.pone.0315910)
Supplement: S1 File — (PDF) [file pone.0315910.s001.pdf]

Appendix 1: STUDY QUESTIONNAIRE
PATIENTS' EXPECTATIONS AND PERCEPTIONS ON QUALITY OF CARE AT A LEADING NATIONAL TERTIARY HOSPITAL IN KENYA

PART A: RESEARCH QUESTIONNAIRE

Introduction and Confidentiality Clause: This research is being carried out by Patient Affairs Department for the purposes of improving the quality of services our customers receive. It is in no way meant to embarrass you or seek details of your condition and treatment. Your identity will remain anonymous and confidential. Part A of the questionnaire will be on expectations and will be administered prior to receiving services while part B will be on perception after receiving services.

DEMOGRAPHICS

Tick as appropriate:

1. Gender : Male ☐ Female ☐
2. What is your age in years? _________________
3. What is your highest level of education attained?

   Not studied ☐ Primary ☐ Secondary ☐ Certificate ☐ Diploma ☐ Undergraduate degree ☐

   Masters' degree ☐
4. Is this your first-time receiving care at this health facility?

   Yes ☐ No ☐
5. How did you find your way to this health facility?

   I was referred from another facility ☐

   I was referred by a friend or colleague ☐

   I came on my own volition ☐

   Other: Specify________________________________

PART 1: EXPECTATIONS-BEFORE THE SERVICE

1.1. RELIABILITY AND QUALITY OF CARE

Circle or tick response that closely describes the extent of your expectation in regards to reliability and quality of care according to these statements:

	**In regards to reliability I expect …**	**1**	**2**	**3**	**4**	**5**
1.	The speed of the services to be	Very slow	slow	medium	fast	Very fast
2.	That waiting times to be	Very long	long	medium	short	Very short
3.	To be explained to my condition and care plan	Strongly disagree	Disagree	Neutral	Agree	Strongly agree
4.	For my concerns and questions to be explained	Strongly disagree	Disagree	Neutral	Agree	Strongly agree

What are your other expectations in regard to reliability in services you are to receive? ____

1.2. ASSURANCE AND QUALITY OF CARE

Circle or tick response that closely describes the extent of your expectation in regards to assurance and quality of care whereby1=Strongly Disagree, 2=Disagree 3=neutral 4= Agree 5 =Strongly Agree

	**In regards to assurance I expect…**	**1**	**2**	**3**	**4**	**5**
1.	To find expert help and definitive treatment	Strongly disagree	Disagree	neutral	Agree	Strongly Agree
2.	My privacy to be ensured during care for example during physical examination	Strongly Disagree	Disagree	neutral	Agree	Strongly Agree
3.	My information to be kept confidential	Strongly Disagree	Disagree	neutral	Agree	Strongly Agree

4.	To receive all the treatment that I need- medication, dressing, other procedure	Strongly Disagree	Disagree	neutral	Agree	Strongly Agree

In what other ways do you expect your well- being to be assured? ____

1.3. TANGIBLES AND QUALITY OF CARE

Circle or tick response that closely describes your expectation in regards to tangibles and quality of care whereby1=Strongly Disagree, 2=Disagree 3=neutral 4=Agree 5 =Strongly Agree

	**In regards to tangibles I expect …**	**1**	**2**	**3**	**4**	**5**
1.	The environment to be clean and hygienically safe	Strongly Disagree	Disagree	Neutral	Agree	Strongly Agree
2.	The food to be tastefully cooked	Strongly Disagree	Disagree	Neutral	Agree	Strongly Agree
3.	The environment to be visually appealing	Strongly Disagree	Disagree	Neutral	Agree	Strongly Agree
4.	The staff to be neat and distinguishable	Strongly Disagree	Disagree	Neutral	Agree	Strongly Agree

What may your other expectations be in regards to tangibles for example your immediate environment? ____

1.4. RESPONSIVENESS AND QUALITY OF CARE

Circle or tick response that closely describes the extent of your expectation in regards to responsiveness and quality of care whereby1= Strongly Disagree, 2= Disagree 3=neutral 4= Agree 5 =Strongly Agree

	**In regards to responsiveness I expect that…**	**1**	**2**	**3**	**4**	**5**
1.	I will get medical assistance from staff whenever I need it	Strongly Disagree	Disagree	Neutral	Agree	Strongly Agree

2.	Staff will be ready and willing to explain and clarify any concerns I have	Strongly Disagree	Disagree	Neutral	Agree	Strongly Agree
3.	I will be explained to my condition and care process	Strongly Disagree	Disagree	Neutral	Agree	Strongly Agree
4.	I will be explained to the timing of services for example medication time, next appointment	Strongly Disagree	Disagree	Neutral	Agree	Strongly Agree

How else do you expect staff to be responsive during care?

____

____

1.5. EMPATHY AND QUALITY OF CARE

Circle or tick response that closely describes the extent of your expectation in regards to reliability and quality of care

	**In regards to empathy I expect…..**	**1**	**2**	**3**	**4**	**5**
1.	To be addressed using my name	Strongly Disagree	Disagree	Neutral	Agree	Strongly Agree
2.	For the person giving care to introduce themselves	Strongly Disagree	Disagree	Neutral	Agree	Strongly Agree
3.	To be treated in a humane and caring way	Strongly Disagree	Disagree	Neutral	Agree	Strongly Agree
4	For staff to be polite and courteous	Strongly Disagree	Disagree	Neutral	Agree	Strongly Agree

In what other ways do you expect to be shown empathy during care?

____

____

PART 2: PERCEIVED QUALITY- AFTER THE SERVICE

2.1. RELIABILITY AND QUALITY OF CARE

Circle or tick response that closely describes your perceptions in regards to reliability and quality of care according to these statements:

	In regards to reliability I perceived …	1	2	3	4	5
1.	The speed of the services to be	Very slow	slow	medium	fast	Very fast
2.	The waiting times to be	Very long	long	medium	short	Very short
3.	my condition and care plan were explained	Strongly Disagree	Disagree	Neutral	Agree	Strongly Agree
4.	my concerns and questions were explained	Strongly Disagree	Disagree	Neutral	Agree	Strongly Agree

In what other ways did you or did you not perceive reliability in services you received? ____

2.2. ASSURANCE AND QUALITY OF CARE

Circle or tick response that closely describes the extent of your perception in regards to assurance and quality of care whereby1=Strongly Disagree, 2= Disagree 3=neutral 4= Agree 5 = Strongly Agree

	In regards to assurance I perceived that …	1	2	3	4	5
1.	I found expert help and definitive treatment	Strongly Disagree	Disagree	neutral	Agree	Strongly Agree
2.	My privacy was ensured during care for example during physical examination	Strongly Disagree	Disagree	neutral	Agree	Strongly Agree
3.	My information was kept confidential	Strongly Disagree	Disagree	neutral	Agree	Strongly Agree
4.	I received all the treatment that I needed- i.e medication, dressing, other procedure	Strongly Disagree	Disagree	neutral	Agree	Strongly Agree

In what other ways did you perceive that your well- being was assured? ____

2.3 TANGIBLES AND QUALITY OF CARE

Circle or tick response that closely describes the extent of your perception in regards to tangibles and quality of care whereby

1= Strongly Disagree , 2= Disagree 3=neutral 4= Agree 5 = Strongly Agree

	**In regards to tangibles I perceived that …**	**1**	**2**	**3**	**4**	**5**
1.	The environment was clean and hygienically safe	Strongly Disagree	Disagree	Neutral	Agree	Strongly Agree
2.	The food was tastefully cooked	Strongly Disagree	Disagree	Neutral	Agree	Strongly Agree
3.	The environment was visually appealing	Strongly Disagree	Disagree	Neutral	Agree	Strongly Agree
4.	The staff were neat and distinguishable	Strongly Disagree	Disagree	Neutral	Agree	Strongly Agree

In what other ways did you perceive that your immediate care environment and surroundings were of quality?

____

2.4. RESPONSIVENESS AND QUALITY OF CARE

Circle or tick response that closely describes the extent of your perception in regards to responsiveness and quality of care whereby1=Strongly Disagree, 2= Disagree 3=neutral 4= Agree 5 = Strongly Agree

	**In regards to responsiveness I perceived that…**	**1**	**2**	**3**	**4**	**5**
1.	I got medical assistance from staff whenever I needed it	Strongly Disagree	Disagree	Neutral	Agree	Strongly Agree

2.	Staff were ready and willing to explain and clarify any concerns I had	Strongly Disagree	Disagree	Neutral	Agree	Strongly Agree
3.	I was explained to my condition and care process	Strongly Disagree	Disagree	Neutral	Agree	Strongly Agree
4.	I was explained to the timing of services for example medication time, next appointment	Strongly Disagree	Disagree	Neutral	Agree	Strongly Agree

How else do you perceive staff to be responsive during care?

____

____

2.5 EMPATHY AND QUALITY OF CARE

Circle or tick response that closely describes the extent of your perception in regards to empathy and quality of care

	**In regards to empathy I perceived that…..**	**1**	**2**	**3**	**4**	**5**
1.	I was addressed using my name	Strongly Disagree	Disagree	Neutral	Agree	Strongly Agree
2.	The person giving care introduced themselves	Strongly Disagree	Disagree	Neutral	Agree	Strongly Agree
3.	I was treated in a humane and caring way	Strongly Disagree	Disagree	Neutral	Agree	Strongly Agree
4	staff were polite and courteous	Strongly Disagree	Disagree	Neutral	Agree	Strongly Agree

In what other ways do you perceive to be shown empathy during care?

____
